# Unconventional Functions of Mitotic Kinases in Kidney Tumorigenesis

**DOI:** 10.3389/fonc.2015.00241

**Published:** 2015-10-27

**Authors:** Pauline Hascoet, Franck Chesnel, Cathy Le Goff, Xavier Le Goff, Yannick Arlot-Bonnemains

**Affiliations:** ^1^UMR 6290 (IGDR), CNRS, University Rennes-1, Rennes, France

**Keywords:** mitotic kinases, non-mitotic functions, Aurora-A kinase, kidney, tumorigenesis

## Abstract

Human tumors exhibit a variety of genetic alterations, including point mutations, translocations, gene amplifications and deletions, as well as aneuploid chromosome numbers. For carcinomas, aneuploidy is associated with poor patient outcome for a large variety of tumor types, including breast, colon, and renal cell carcinoma. The Renal cell carcinoma (RCC) is a heterogeneous carcinoma consisting of different histologic types. The clear renal cell carcinoma (ccRCC) is the most common subtype and represents 85% of the RCC. Central to the biology of the ccRCC is the loss of function of the Von Hippel–Lindau gene, but is also associated with genetic instability that could be caused by abrogation of the cell cycle mitotic spindle checkpoint and may involve the Aurora kinases, which regulate centrosome maturation. Aneuploidy can also result from the loss of cell–cell adhesion and apical–basal cell polarity that also may be regulated by the mitotic kinases (polo-like kinase 1, casein kinase 2, doublecortin-like kinase 1, and Aurora kinases). In this review, we describe the “non-mitotic” unconventional functions of these kinases in renal tumorigenesis.

## Introduction

Renal cell carcinoma (RCC) represents approximately 3.8% of adult malignancies and 90–95% of kidney neoplasms. The most common histological RCC subtype is the clear cell carcinoma (ccRCC), which accounts for 85% of cases. At least 80% of ccRCCs have deletions or translocations involving the short-arm of chromosome 3, which contains the von Hippel–Lindau (VHL) gene at 3p25. Clear cell carcinoma shows highly consistent chromosomal aberrations involving loss of the short-arm of chromosome 3, a partial or complete trisomy 5q, and abnormalities of 6q, 8p, 9p, 10q, 11q, 12q, and 14q. The second most common cytogenetic abnormality associated with ccRCC is a gain of chromosome 5q6; however, very little is known about its effects. Mitotic errors and misregulation of cell-cycle process are considered to be an important characteristic of kidney cancer. Successful cancer therapies depend mainly on the recognition of physiologic targets that are primarily involved in the regulatory mechanism of cell-cycle progression (1). The members of serine/threonine kinases, such as cyclin-dependent kinases, polo-like kinases, and aurora kinases, are the well studied families that coordinate the mitosis sequence (2). Many studies are generally focused on the role of mitotic functions of these kinases and efforts have been put up to use targets for generation of new anti-cancer drugs (3). However other non-mitotic functions of these kinases have been identified as diverse as control of the resorption of the cilia, the cell differentiation, and the cell polarity control in interphase cells.

### Kidney Cancer and (Loss of) Cell Polarity

Carcinomas are frequently characterized by loss of cell differentiation and excess of cell proliferation. They are also characterized by a loss of cell polarity, which includes polarity complexes and adhesion complexes proteins dysfunction (4–6). Loss of cell polarity induces cancer development by deregulating different signaling pathways (7). Ultimate loss of the epithelial phenotype may contribute to epithelial-to-mesenchymal transition (EMT) and metastasis. Cell polarity is defined as asymmetry in functional organization of the cell. It is required for the formation and maintenance of functional epithelia. Epithelial cells are highly polarized (i.e., asymmetric distribution of lipids, proteins, RNA, organelles) and are tightly connected by specialized intercellular junctions. In epithelia, maintenance of apico-basal (AB) polarity is crucial for preserving epithelial integrity and depends on cell polarity complexes and cell junctions. Cell polarity is mostly driven by complexes composed of adherens junctional complexes (including cadherin and catenin) and tight junctional complexes that contain zonula occludens (ZO) proteins, occludin, claudin, and junctional adhesion molecules (JAM). These cell polarity complexes have antagonistic roles in regulating the specific distribution of key molecules. The Scribble complex (Scrib, Dlg, and Lgl2) is present in the baso-lateral region of the cell and has antagonistic roles with the apical complexes, Par (Par3, Par6, Cdc42, atypical protein kinase C) and Crumbs (Crb3, Pals1, and PatJ). Other conserved Par proteins such as Lkb1 (Par4) and the Par1 kinase regulate cell polarity (8).

In the kidney, the epithelium is a simple tube-based structure with the apical membrane facing the lumen whereas the basolateral membrane interacts with the matrix. In kidney tubes, as well as for podocytes, the establishment and maintenance of the epithelium require the presence of the Par, Crumbs and Scribble complexes (9). AB polarization of the renal epithelium is crucial for the appropriate function of the normal kidney in waste products and extra water removal and electrolyte balance. Disruption of cell polarity in kidney is involved in renal pathologies such as acute renal failure, which affects tight junction function (10) and polycystic kidney disease (PKD) in which polarity factors are frequently mislocalized (11). Polarity complexes expression and functions are frequently deregulated in cancer. Implication of polarity complex proteins as oncogene or tumor suppressor in tumorigenesis depends on the kind of alteration of their expression. It is also dependent on the origin of the epithelium (12). For instance, Par3 expression is lost in glioblastoma, esophageal squamous carcinoma, breast, lung, head, and neck cancers (13–16) but is overexpressed in some RCC (17) or in some severe hepatocellular carcinoma (18). Thus both up- and down-regulation of critical cell polarity proteins may be associated with tumorigenesis. Consistent with this notion, expression and functional studies in different skin tumors showed either oncogenic or tumor-suppressive functions of Par3 depending on the cellular context (19).

It has been widely admitted that ccRCC originates from proximal epithelial tubular cells. This is based on specific cellular marker expression (20, 21) or genetic expression pattern (22). However, epithelial cells of other nephron segments may also promote some cases of ccRCC. Interestingly, based on studies in VHL patients, ccRCC may also arise from VHL-null epithelial cells, which show markers of dedifferentiation (23). In colon adenocarcinoma, it has been reported that both expression levels and subcellular localization of the Scribble polarity complex component Dlg could be deregulated. However, in ccRCC, Dlg expression level is unaltered compared to normal kidney epithelial cells, but the protein is mislocalized in a granular distribution in clear cells. It was proposed that altered localization of Dlg may contribute to cell transformation and promotes high migration ability (24).

In ccRCC, the polarity protein Par3 overexpression is correlated with a poor prognosis (17). The localization of Par3 at both the plasma membrane and in the cytoplasm was associated with worse clinical factors in a cohort of 101 ccRCC patients (25). Furthermore, Dugay et al. showed that Par3 up-regulation was associated with cytoskeleton defects and increased cell migration capacity. This was restored by Par3 down-regulation whereas other Par complex component expression levels remained unchanged (17). Crumbs3 (Crb3) is involved in establishment of AB polarity and formation of tight junctions. Knock-out mice showed strong defects in epithelial AB polarity and thus in establishment and maintenance of epithelial cells. In contrast to Par3, loss of Crb3 expression induces tumorigenesis in kidney cells. This effect was associated with several characteristics of cell polarity disruption. This phenotype was reversed upon restoration of Crb3 expression (26, 27). Accordingly, loss of Crb3 is associated with a shorter overall survival in ccRCC (28). Altogether, polarity factors mislocalization and/or their deregulated expression are associated with tumor progression. This may be due to altered epithelia organization involving cell–cell contact disruption, a higher ability to transform phenotype (EMT) and for cell migration, and/or increased cell proliferation signaling.

### Kidney Cancer and EMT

The EMT is known for more than a decade to participate in tumor progression and metastasis formation in many carcinomas. This process by which epithelial cells acquire a mesenchymal phenotype starts with loss of cell–cell adhesion and polarity and leads to increased cell motility and invasion. At the molecular level, EMT is characterized by the alteration of some gene expression profiles resulting in down-regulation of epithelial markers (such as E-cadherin, ZO-1, and cytokeratins), and up-regulation of mesenchymal markers, such as vimentin (29).

The implication of EMT in clear cell renal cell carcinoma (ccRCC) progression and invasiveness has been established within the last 5 years, especially when investigators were searching for new biomarkers associated with clinical outcome. While comparing mRNA levels of 46 EMT-related genes between RCC and healthy kidney samples, Chen and colleagues showed that low vimentin, CXCR4, fibronectin, and TWIST1 transcript levels are correlated with a better outcome whereas overexpressed vimentin and CXCR4 constitute independent markers for poor prognosis in RCC patients (30). Likewise, using an immunohistochemical approach on a cohort of 122 RCC patients, protein expression levels of vimentin, TWIST, E-cadherin, and clusterin were identified as predictors of disease recurrence (31). Overexpression of zinc-finger transcription factors SNAI1/Snail or ZEB2/SIP1, well-known EMT inducers acting as repressors of E-cadherin transcription, has also been associated with poor prognosis in RCC patients (32, 33). It could result at least in part from the loss or a decreased expression of NDRG2 and FOXO3a observed in high grade or metastatic RCC tumors, respectively (34, 35). Several mechanisms have been proposed to explain how EMT is induced and/or sustained during kidney tumorigenesis.

As a consequence of the functional inactivation of VHL very often observed in ccRCC, accumulation of Hypoxia-induced transcription factors HIF1 and/or 2α leads to the transcriptional activation of many HIF target genes. Among them, erythropoietin (EPO) may stimulate EMT in RCC *via* the PI3K/Akt/mTOR pathway (36) in agreement with the observations that the mTOR inhibitor Everolimus was able to slow down RCC tumor growth and to reverse EMT phenotype in a mouse xenograft model (37). At least two proinflammatory cytokines, tumor necrosis factor alpha (TNFα) and interleukin 15 (IL-15), have been reported to play a role in EMT induction in RCC. Serum levels of TNFα are significantly increased in RCC patients as well as secretion by tumor cells and it was demonstrated that TNFα promotes EMT in RCC by decreasing E-cadherin expression and increasing vimentin expression and MMP9 activity (38, 39). This TNFα effect is mediated through the inhibition of GSK3β in a NFκB-independent manner (39, 40). In contrast, IL-15 production is not altered in RCC but the IL-15 signaling pathway is profoundly modified because of the expression of a particular transmembrane IL-15 form and the defective expression of CD132 (γc chain of the IL-2 receptor family) and JAK3 (41), which both favor EMT through down-regulation of E-cadherin expression (42).

### Kidney Cancer and Angiogenesis

To proliferate, cancer cells require a continuous supply of nutrients and oxygen. This supply is function of the distance between tumor vessels and cancer cells, leading to intratumoral hypoxia heterogeneity (43). To overcome this phenomenon, angiogenesis is up-regulated in most cancers, due to an overproduction of angiogenic stimulators and the consequent unbalanced proportion of activators (such as VEGF, MMPs, FGF, HGF…) and inhibitors (such as thrombospondins, endostatin, angiostatin…) in favor of the hyperactive tumor vasculature development (44). Therefore, tumor vessels are disorganized, tortuous, and mal-shaped with fewer mural pericytes (45). Moreover, proliferating cancer cells can also exert a pressure on intratumoral blood and lymphatic vessels (46) leading to an impaired blood perfusion. These vascular abnormalities lead to a hypoxic, acidic, and hypertensive tumor microenvironment. Adaptation to hypoxia at the cellular level is mainly regulated by the Hypoxia Inducible Factors (HIFs).

In ccRCC, the HIF system is up-regulated both by micro-environmental hypoxia and a genetic event, the VHL inactivation, that lead to HIF-α stability. Indeed, the *VHL* gene is deleted, mutated or hypermethylated in ~90% of the cases, leading to the absence or to the expression of a non-functional pVHL protein (47). HIFs, the main targets of pVHL, are transcription factors responsible for numerous hypoxia responses by promoting expression of genes involved in the cellular adaptation to hypoxia. In normoxia, the oxygen-dependent prolyl hydroxylated domain containing proteins (PHDs) specifically hydroxylate HIF-α in its N-terminal transactivation domain (NTAD) (48, 49), allowing its interaction with pVHL, the substrate recognition subunit of an E3 ubiquitin ligase complex, and its subsequent degradation by the 26S proteasome (50). HIF-α can also be hydroxylated in an oxygen-dependent manner on its C-terminal transactivation domain (CTAD) by the Factor Inhibiting HIF (FIH-1). This hydroxylation prevents the recruitment of the transcriptional co-activators CBP and p300. Thus, under low oxygen conditions or in absence of a functional pVHL, HIF-α is stabilized and can dimerize with the stable HIF-β, and this heterodimer transcriptionally activates up to 200 genes involved in cell growth, glucose metabolism, angiogenesis, apoptosis, pH regulation… One of the most described targets of HIFs is the vascular endothelial growth factor (VEGF). It is overexpressed at the mRNA and protein levels in ccRCC compared to normal kidney tissues (51, 52). Endothelial cells but also RCC cells express the VEGF Receptor (VEGFR-2), inducing increased tumor angiogenesis. VEGF and its receptor constituted the main targets for metastatic RCC treatments such as Sunitinib, Sorafenib, Pazopanib, and Bevacizumab (53–55). However, most of the ccRCC patients develop resistance to these VEGF inhibitors. mTOR inhibitors (Temsirolimus and Everolimus) were also used for RCC treatment by acting downstream of the VEGF receptor through HIF down-regulation since mTORC1 drives HIF-1α synthesis (56).

The HIFα family is composed of three different members (HIF-1α, HIF-2α, and HIF-3α). HIF-1α is ubiquitously expressed whereas HIF-2α expression is more restricted, but both isoforms are co-expressed in numerous cell types. HIF-1α and HIF-2α have common and also unique targets and are thought to have overlapping functions, but also divergent outcomes in tumorigenesis. In fact, HIF-1α and HIF-2α were first considered as essential for ccRCC progression but several studies tend toward an oncogenic role for HIF-2α in ccRCC and a tumor suppressor function for HIF-1α (23, 57, 58). Moreover, most of the VHL^−/−^ ccRCC cell lines do not express HIF-1α whereas they all express HIF-2α, suggesting a selective pressure to maintain HIF-2α expression.

### Kidney Cancer and Ciliogenesis

Kidney epithelial cells have developed primary cilia that extend into the tubular lumen. This includes cells in the parietal layer of Bowman’s capsule, proximal tubules, the loop of Henle, and the collecting duct. Cilia are present on almost all cells lining the nephron, with the exception of intercalated cells (59).

In the kidney, the cilium serves as a flow sensor in the kidney tubules lumen, with flow-induced ciliary bending causing a transient increase in intracellular calcium (60). Polycystins (PC) 1 and 2, gene products of PKD1 and PKD2, are large multi-pass transmembrane proteins of the transporter receptor potential channel (TRPC) family of calcium transporters. Mutations of PKD1 and PKD2 induce cyst formation in the kidney, and cyst formation in part arises because of derestricted cell proliferation. Thus PC1 and PC2 at least indirectly participate in cell-cycle control. Signaling downstream of PC1 and PC2 is quite complex. In normal cells, these proteins negatively regulate the cAMP and Raf–MEK–ERK signaling pathways (61), both of which being hyper activated in renal epithelial cysts (62).

The primary cilium of the kidney epithelium mediates sensation of mechanical signals produced by apical fluid shear stress and its transduction into an intracellular Ca^2+^ signaling response (63, 64). In cell culture, deflection of the cilia axoneme initiates a transient increase in the level of intracellular Ca^2+^ resulting from Ca^2+^ entry through a channel, possibly PC1 and PC2, located in the cilium. This is supported by the fact that the Ca^2+^ influx generated by fluid flow is abolished in cell lines lacking polycystin-1 (65) and by the loss of fluid flow-mediated calcium signaling in the embryo.

Accordingly, defects in assembly or function of primary cilia lead to a plethora of developmental disorders and pathological conditions known as ciliopathies (66). Cystic kidney disorders are one of the leading causes of end-stage renal disease. Several proteins implicated in the pathogenesis of PKD localize to cilia. In the growth of the renal normal tubule, the mitotic spindle of dividing cells aligns along the axis of the nephron. However, in cells with mutations within *Pkhd1* (encoding fibrocystin) as well as *Hnf-1* genes, the spindle fails to correctly orient inducing abnormal cell division (67).

An important feature of ciliary signaling is the continuous interaction with regulatory signaling molecules at the ciliary base, i.e., the centrosomal region, which may coordinate the crosstalk between separate ciliary signaling pathways to activate specific cellular targets and gene arrays for specified cellular or tissue responses (67, 68).

The VHL disease is caused by germline mutation in the *vhl* tumor suppressor gene. One of the major clinical manifestations of the disease includes kidney tumor. Around 80% of the VHL patients developed renal cysts. pVHL localized to the cilia. Ectopic expression of VHL gene in renal clear cell carcinoma cell lines restored cilia formation, implying that pVHL might directly support ciliogenesis (69). Inactivation of VHL and GSK-3b was required to allow loss of cilia based on cooperative function of these proteins in ciliary maintenance. pVHL also regulates microtubule orientation during ciliogenesis and interacts with the Par3–Par6-atypical PKC complex, which supports ciliogenesis (70). Moreover, pVHL associates with kinesin 2, allowing pVHL to influence microtubule dynamics in support of cilia (71, 72).

### Genetic Instability and Kidney Cancer

A large proportion of tumors are aneuploid and this abnormal number of chromosomes is thought to contribute to tumorigenesis. It is associated with poor prognosis, multidrug resistance, and increased capacity to metastasis. Genomic instability may be the result of several mitotic defects (73–75).

During mitosis, the formation of a bipolar mitotic spindle is essential for proper chromosome segregation. The chromosome segregation mediated by the anaphase-promoting complex/cyclosome (APC/C) is controlled by the spindle assembly checkpoint (SAC). This checkpoint arrests cell-cycle progression by modulating the activity of the mitotic kinase CDK1 until all chromosomes are properly attached to the mitotic spindle (76, 77). The chromosomal passenger complex (CPC), composed of Aurora-B, INCENP, Borealin, and Survivin, plays also a critical role at different stages of mitosis and cytokinesis by recruiting condensin complex in mitotic entry (78, 79) and by activating SAC in metaphase if chromosomes are not properly attached to the spindle. Its relocalisation from anaphase chromosomes to cell equator promotes mitotic exit and cytokinesis (80). Hence, deregulated CDK1 activation, weakened mitotic checkpoint, defective chromatid cohesion or condensation, may contribute to genomic instability. Indeed several alterations in mitotic genes have been reported in human cancer with chromosome instability (81). The duplication and maturation of centrosome is a critical step for the formation of a bipolar mitotic spindle. Thus, centrosome amplification, enrichment for centrosome components [such as CDK1, NEK2, Aurora-A, Aurora-B, or Polo-like kinase 1 (Plk1)] or merotelic attachment of chromosomes to the mitotic spindle may promote abnormal cell division and aneuploidy (77, 82, 83).

A recent study on VHL showed that this protein is a controller of mitotic fidelity *in vivo* (84). VHL is localized to the mitotic spindle. The loss of function of pVHL *in cellulo* and *in vivo* provokes spindle misorientation as a result of unstable astral MT and chromosome instability due to SAC impairment (85, 86). This aneuploidy phenotype in VHL-deficient renal carcinoma cell lines is suppressed by ectopic expression of Mad2, a regulator of APC/C (85) or inhibition of miR-28-5p, a key regulator of Mad2 protein translation upregulated in a variety of cancers (84, 87). This result suggests that low level of Mad2 is linked to chromosomal instability (CIN) in VHL-associated kidney cancers (86).

It has been observed that the VHL loss of function is not sufficient to explain tumor formation in kidney. Recently, Albers et al. have shown that secondary genetic alterations of p53 can cooperate with loss of pVHL to induce tumors in mice (88). Detailed cytogenetic analysis of tumor at different stages and gene expression analysis are necessary to understand tumor development and the molecular basis that contribute to aneuploidy.

### Non-Mitotic Roles of Mitotic Kinases in Kidney Cancer

#### Polo-Like Kinase 1

Polo-like kinase 1 is the best characterized member of the Plks family (89). This serine/threonine kinase is known to regulate multiple stages of mitosis. Its expression is cell-cycle regulated since it increases from late S phase to mitosis, and its degradation mediated by APC/C starts in late mitosis. In interphase, Plk1 is expressed in the cytoplasm and at the centrosomes whereas in mitosis, it localizes to the centrosomes in prophase, then becomes enriched at kinetochores during prometaphase and metaphase, and in late mitosis a fraction of Plk1 is found at the spindle midzone (90). Plk1 is not only involved in the assembly of a bipolar spindle, centrosomes duplication and maturation, DNA replication, and DNA damage checkpoint, but also in the control of the G2/M transition. Several studies demonstrate its role not only in sister chromatid dissociation but also in mitotic exit and cytokinesis [reviewed in Ref. (91, 92)].

Since Plk1 is a key regulator of the cell division, it evidently appeared as an interesting target for anti-mitotic chemotherapeutic drugs (93). Small-molecule inhibitors of Plk1 activity have been developed. Several phase II trials were performed in solid tumors using for instance BI2536, a Plk1 inhibitor, but it exhibited a limited anti-tumoral activity, suggesting that more favorable pharmacological derivates are required (94). Despite these non-conclusive trials, Plk1 remains a promising target since it is overexpressed in a number of human tumors: esophageal squamous cell carcinoma (95), hepatocellular carcinoma (96), bladder carcinoma (97), thyroid carcinoma (98, 99), colorectal cancer (100), pancreatic cancer (101), prostate cancer (102), melanoma (103), breast cancer (104), and ovarian cancer (105). Its overexpression is often correlated with tumor grade, sometimes with metastatic disease (103), and proposed as prognosis factor. Interestingly, a gene expression profiling performed on ccRCC patient primary tumors identified Plk1 as significantly correlated with disease malignancy (106). Plk1 is overexpressed at the mRNA and protein level in RCC patient tissues (107) and this overexpression is correlated with the tumor grade and metastases. Moreover, Plk1 knock-down by siRNA strategy or small-molecule inhibitor decreased ccRCC cell proliferation *in vitro* by G2/M blockade (106) and invasion properties (107). To date, no clinical trial was performed using Plk1 inhibitors alone or in combination with other drugs in RCC patients. Intratumoral injections of Plk1 inhibitor in ccRCC xenograft nude mice induced a tumor volume decrease indicating that a sustained inhibition of Plk1 function may inhibit ccRCC tumor growth *in vivo* (106). Intriguingly, another *in vivo* study reported that a liposomal anti-Plk1 siRNA delivery system failed to inhibit tumor growth in a mouse xenograft RCC model (108).

Non-mitotic functions were recently attributed to Plk1, including a role in cilia disassembly. Indeed, an overexpression of Plk1 dramatically reduces the length and the percentage of primary cilia whereas the depletion of Plk1 or the inhibition of its kinase activity induces a delay in cilia disassembly (109). Even if Plk1 is not required for proper ciliogenesis (110), Lee and colleagues identified a new primary cilia disassembly pathway mediated by Wnt5a, CK1ϵ, Dvl2, and Plk1 (111). Under growth stimulation, the non-canonical ligand of the Wnt pathway, Wnt5a, enhances the Plk1 bound to Dvl-2, formerly phosphorylated by CK1ϵ. This interaction stabilizes HEF1 and the HEF1/AurA complex, leading to the HEF1/AurA dependent primary cilia disassembly. Another study has demonstrated that Plk1 is recruited to the pericentriolar matrix by PCM1, a centriolar satellite protein (109). This interaction requires prior phosphorylation of PCM1 by CDK1. Then, Plk1 is activated by Aurora-A and promotes primary cilia disassembly. Moreover, KIF2A, a member of the kinesin-13 protein family with only an ATP-dependent microtubule depolymerization activity, is phosphorylated by Plk1 at the level of subdistal appendages of the mother centriole (112). This event enhances MT depolymerization to disassemble primary cilia. KIF24, another kinesin-13 protein, was previously described to suppress inappropriate ciliogenesis in proliferating cells by stabilizing CP110 (113). In quiescent cells, KIF2A and KIF24 are both ubiquitinated by APC/C, which may prevent a premature initiation of cilia disassembly (112). Interestingly, this Plk1–KIF2A pathway was found constitutively active and described as involved in defective ciliogenesis in a ciliopathy named premature chromatid separation (PCS) syndrome (114). Of note, Plk1 was also associated to another ciliopathy, the nephronophthisis (NPH), a cystic kidney disease. Indeed, Plk1 colocalizes at the transition zone of the cilia with nephrocystin 1 (NPHP1), a scaffold protein of the NPH protein complex frequently mutated in NPH patients. Plk1 phosphorylates NPHP1, leading to cilia disassembly (115). The function of this phosphorylation and its link with cilia disassembly remain to be determined, but this study highlighted another signaling role for Plk1 in cilia disassembly at the transition zone. Further investigations are needed to study whether the non-mitotic function of Plk1 in cilia disassembly is involved in ccRCC development since this pathology is strikingly linked to cystic lesions and thus cilia defects.

#### Casein Kinase 2

Casein kinase 2 (CK2) is a ubiquitously expressed and much conserved serine/threonine protein kinase, which is composed of two catalytic α- (or α′-) and two regulatory β-subunits. This constitutively active kinase is involved in cell proliferation and survival; it exerts pleiotropic effects throughout cell-cycle progression and notably during mitosis, when CK2α is transiently hyperphosphorylated (116). During the G2-M transition, it first plays a role in chromatin condensation by phosphorylating DNA topoisomerase 2α (117). It also participates in the activation of M-phase promoting factor, CDK1-cyclin B, both by phosphorylating/activating the phosphatase CDC25B and by facilitating PLK1-mediated Wee1 inhibition (118, 119). CK2 is then located on the mitotic apparatus in a Pin-1-dependent manner where it shares with Plk1 the microtubule plus-end-tracking protein CLIP170 as a substrate, the phosphorylation of which regulates the timely microtubule-kinetochore attachment and contributes to proper chromosome alignment at metaphase (120). Together with the Aurora-B mitotic kinase, CK2 is later involved in spindle elongation and chromosome segregation during anaphase in yeast and interestingly, loss of CK2 activity has been shown to activate SAC (121).

**Figure 1:**
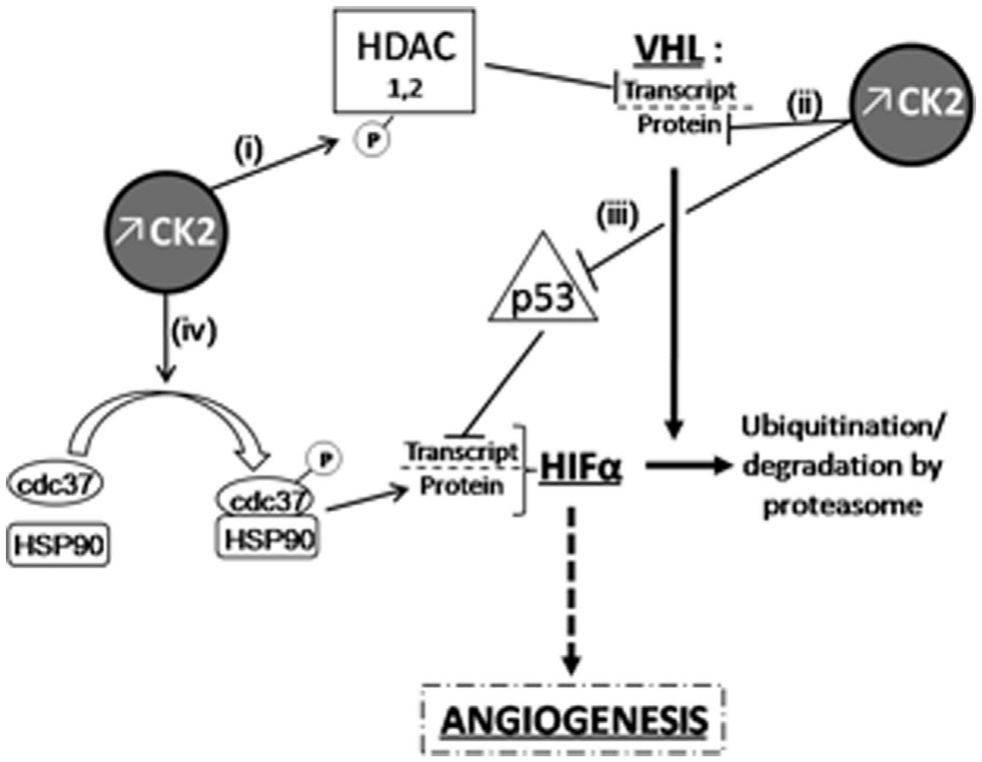
**Overexpressed CK2 induces HIFα accumulation through VHL-dependent or independent mechanisms**. CK2 can down-regulate VHL expression at the transcriptional level (i) by phosphorylating HDACs (125) as well as at post-translational level and (ii) by destabilizing phosphorylation in the NH_2_-terminal acidic domain of VHL (126). In the latter case, CK2-induced destabilization of VHL also results in p53 inactivation, which can no longer inhibit HIFα transcription (iii); (126, 127). Finally, (iv) cdc37 phosphorylation by CK2 allows HSP90/cdc37 dimer formation and the subsequent interaction of this complex with HIF-1α, which is essential for its cellular stability (128, 129).

Casein kinase 2 has been shown to be overexpressed, and its activity increased, in ccRCC compared to healthy kidney (122), as reported in many other cancer types. Such deregulation of CK2 activity during mitosis may be sufficient to cause mitotic chromosome instability and the ensuing aneuploidy that have been established to promote tumorigenesis. There is however evidence from several reports regarding solid tumors to suggest that overexpression of CK2 could also influence kidney tumor development through non-mitotic mechanisms. First, CK2 could exacerbate angiogenesis in hypoxic renal tumors since the potent and selective small molecule CK2 inhibitor CX-4945 (also called silmitasertib) has been shown to exert anti-tumor activity through inhibition of angiogenesis in breast cancer (123). This proangiogenic effect of CK2 has been recently discussed [see for review in Ref. (124)]; it is likely the result of several possible actions of the kinase in the signaling cascade leading to hypoxia-mediated transcription of HIF target genes (see Figure 1 for details).

Proangiogenic effect of overexpressed CK2 could additionally be mediated by local overproduction, by tumoral and peripheral blood mononuclear cells, of prostaglandin E2 (PGE2), the most predominant and biologically active eicosanoid produced by cyclooxygenase 2 (COX-2); this enzyme is indeed often upregulated in ccRCC, likely through a CK2-induced Wnt/β-catenin pathway and its expression is correlated with prognosis [see for review in Ref. (130)].

Besides, overexpression of COX2 is also associated with tumor cell migration and invasion as well as the PGE2 receptor EP4, the expression of which is strongly correlated with ccRCC tumor stage and aggressiveness and the presence of metastases (130) but there is to date no such available data connecting CK2 expression levels and tumor progression. A few recent studies on other solid tumors (lung and colon) nevertheless suggest that CK2 could also be involved in the EMT: CK2α is overexpressed in colorectal cancer compared to adenoma or healthy colorectal tissue and its silencing by RNA interference, while slowing down tumor cell proliferation also inhibited cell motility and invasion. At the molecular level, CK2α knock-down was able to reverse the EMT process by decreasing vimentin expression and upregulating E-cadherin (131). Similar observations were reported in A549 lung adenocarcinoma cells in which CK2 inhibition with CX-4945 strongly decreased cell migration and invasion at least through the attenuation of PI3K/AKT and ERK signaling pathways and blocked TGF-β-induced EMT (132, 133). Further investigation is worth pursuing in ccRCC to reveal such implication of overexpressed CK2 in EMT process and in the regulation of tumoral cell migration and invasion.

#### Doublecortin-Like Kinase 1

Doublecortin-Like Kinase 1 (DCLK1) was first isolated in *Caenorhabditis elegans* as the product of the zyg-8 gene. Zyg-8 loss-of-function alleles were isolated in a visual screen for mutants deficient in spindle positioning in the *C. elegans* one-cell stage embryo. The phenotype was due to shortening of astral MT during anaphase leading to a defect in spindle-cortex interaction. ZYG-8 is a microtubule-associated protein (MAP), which promotes MT stabilization. ZYG-8 encodes a protein kinase that harbors a catalytic kinase domain and a domain similar to human Doublecortin (DCX), a MAP, which stimulates MT polymerization. Both domains are required for its function in *C. elegans* where ZYG-8 localizes to MT. ZYG-8 kinase also binds to MT *in vitro* and *in vivo* through its DCX domain in mammalian cells. ZYG-8 function is required to maintain spindle architecture during anaphase and influences spindle positioning and thus the proper progression of mitosis (134). Later, it was shown that DCLK is a MT-associated kinase, which regulates spindle formation in neuronal cells. DCLK overexpression induced large monopolar spindles and a prometaphase arrest in mitotic neuronal cells. On the other hand, DCLK silencing perturbed mitotic spindle organization with shorter and thinner MT also promoting prometaphase arrest (135). Thus, in metazoans, DCLK1 has a conserved mitotic function in regulating spindle formation.

Doublecortin-Like Kinase 1 was identified as a marker of tumor stem cells (TSC) for instance in pancreatic and colorectal cancers (136) and is upregulated in many other solid tumors. It has been recently suggested that DCLK1 may constitute a potential relevant diagnostic and prognostic marker of circulating cancer cells [e.g., pancreatic adenocarcinoma (137)]. RCC cells share many characteristics with TSC, including an EMT phenotype. Interestingly, DCLK1 is epigenetically dysregulated and overexpressed in more than 90% of RCC tumors. Increased expression of DCLK1 correlates with stages II and III tumors. Expression of DCLK1 was correlated with the EMT phenotype in RCC. Consistently, silencing of DCLK1 in renal Caki-2 cell line promoted EMT-specific transcription factors down-regulation (SNAI1/SNAI2, TWIST1, and ZEB1) and reduced migration and invasion capacities. A decreased adhesive phenotype was also observed and correlated with a decrease in the expression of the focal adhesion regulator PTK2 (FAK). DCLK1 may regulate migration and invasion through the maintenance of focal adhesion (138). Whether MT and F-actin binding of DCLK1 is involved in its adhesion function remains to be investigated. Interestingly, it has been recently suggested that phosphorylation of FAK by the sphingosine kinase-1 may promote renal cell invasion (139). Thus DCLK1 may contribute to the metastatic process and targeting this kinase should be considered as part of an anti-cancer therapy (140).

#### Aurora-A Kinase

Aurora-A is a centrosomally localized cell-cycle regulatory serine/threonine kinase that activates the cyclin B1-Cdk1 mitotic kinase and coordinates formation of a bipolar spindle and nuclear envelope breakdown in M phase. The serine/threonine kinase Aurora-A localizes on duplicated centrosomes from the end of S phase to the beginning of the following G1 and is essential for mitotic entry, centrosome duplication, spindle formation, chromosome segregation, and cytokinesis (141). Aurora-A is activated by phosphorylation from the end of S phase until the next G1 when the kinase is ubiquitinated and degraded by the proteasome in a Cdh1-dependent manner (142, 143). The major roles of Aurora-A kinase have been widely described in the centrosome separation and spindle formation. Aurora-A is activated mainly by Ajuba (144); TPX2, Bora, as well as protein phosphatase inhibitor-2 (145, 146) and the focal scaffolding proteins HEF1 and NEDD9 (72). Phosphorylation of Aurora-A on T288 residue within the activation loop of the catalytic domain results in the activation of the protein. Aurora-A has multiple other phosphorylation sites modulating its mitotic and non-mitotic activities (S51, S53/S54, S66/S67, S89, S98, and S342 residues) (147). The relevance of the different phosphorylation sites is not actually well known.

The human *Aurora-A* gene resides at chromosome 20q13.2, a region that is commonly amplified in primary breast tumors, colorectal cancers, and other cancer cell lines, including breast, ovarian, colon, prostate, neuroblastoma, and cervical cell lines (148). Aurora-A abnormalities have been reported in a variety of malignant tumors correlated with an *Aurora-A* gene amplification or upregulation of Aurora-A expression in tumor tissues compared with normal tissues (149). Controversial studies suggested that Aurora-A abnormalities were positively correlated with aggressive tumor behavior invasion, and nodal metastasis, but some studies showed no correlation or an inverse correlation (150). The oncogenic effects of Aurora-A was attributed to its interaction with several important cellular proteins, including protein phosphatase 1, target protein for xklp2, HEF1, p53, CENP-A, Ajuba, and transforming acidic coiled-coil (141).

Even though the expression of the kinase from S to M phase and the localization of Aurora-A onto the centrosome and the spindle pole have been widely described, it becomes more and more evident that Aurora-A is present in all phases of the cell cycle and might fully participate in other functions that mitotic ones. Several recent studies have described non-mitotic functions of Aurora-A in cellular calcium signaling, cilia resorption or cytoskeleton organization. The presence of diffuse Aurora-A staining in the cytosol, the Golgi and perinuclear region hinted that it had a possible role unrelated to mitosis (151).

Previous studies have shown a potential role of Aurora-A in metastasis and that Aurora-A ectopic expression induced a robust increase in cell migration through its effect on tubulin polymerization (147, 152). The mechanism by which the kinase is involved in the process of mobility, migration, and invasion is not completely defined but it has been suggested a role of RAS, AKT, RALa, and MAPKs (153). Aurora-A has been shown to be implicated in cell migration along with SRC and FAK and, unexpectedly, is regulated by a lipase, phospholipase D2 (PLD2). Phosphatidic acid is able to bind and activate Aurora-A causing rapid tubulin polymerization and leading to an enhanced cell migration (154). The action of Aurora-A on cell mobility has also been investigated through a group of proteins involved in the focal adhesion as NEDD9, SRC and FAK. The effect of Aurora-A on cell migration is augmented in the presence of SRC and, in return, Aurora-A also activates FAK. Cell migration is also physically mediated by actin cytoskeleton and is initiated by the protrusion of the cell membrane (155). Overexpression of Aurora-A regulates actin reorganization, leading to free barbed end formation. Cofilin has emerged as an essential player for the localized formation of the barbed ends, which act as sites for new local actin polymerization, thus determining the direction of cell protrusion and movement (156). A significant correlation between Aurora-A expression and cofilin dephosphorylation was described in the immunohistochemical analysis of clinical breast cancer specimens, supporting a novel signaling mechanism by which Aurora-A indirectly induced cofilin dephosphorylation and actin reorganization, thus promoting mammary cell movement and breast cancer metastasis (157). Most importantly, Aurora-A was demonstrated to enhance EMT and invasiveness *via* activation of MAPK signaling pathway. A novel oncogenic cross-talk between Raf/MAPK and Aurora-A signaling pathways has been established in the development of EMT, stemness, and tumor progression in ERα breast cancer cells. The constitutive activation of MAPK signaling pathway during tumor growth leads to the stabilization and accumulation of Aurora-A which induces the EMT (158). Aurora-A inhibition by VX-680 induced a significant suppression of cell invasion ability, as well as reversed its EMT behavior by reducing membrane expression of epithelial markers E-cadherin and β-catenin in cervical CN2 cells (152, 159).

Recent studies have shown more diverse, non-mitotic functions of Aurora-A orchestrating remodeling of the microtubular cytoskeleton during neurite extension (160) but also regulating protrusion and resorption of cellular cilia participating in cellular calcium signaling (161, 162). Aurora was shown to localize at the basal body of the cilium and its activation was shown to participate to the ciliary resorption by promoting histone deacetylase-dependent tubulin depolymerization of the ciliary axoneme (161).

The serum growth factors were also shown to induce Aurora-A activation at the basal body of the cell cilium in non-cycling G0/G1 cells causing Aurora-A and NEDD9-dependent cilia resorption (161). HEF1/Cas-L/NEDD9 is a component of focal adhesions that has a prominent role in inducing metastasis and that co-localizes with Aurora-A at the centrosome, thereby enhancing the effect of Aurora-A on the resorption of the cilium. After cilia resorption, Aurora-A ceases to be active (as judged by kinase activity) and will probably be reactivated for mitotic function as soon as the cell enters the cell cycle. By microinjecting active Aurora-A into cells, the cilium disappeared, leading to the conclusion that active Aurora-A is necessary and sufficient to induce cilium resorption (163). Loss of cilia associated with high level Aurora-A expression would indirectly impact the functionality of the cilia-dependent and cancer-relevant signaling cascades, such as those involving Hedgehog (164).

Pathological conditions of the kidney include renal cell carcinoma, in which elevated Aurora-A expression has (often) been reported (165) as well as its partner NEDD9 (72, 166, 167). Aurora-A pathway is induced through HIF (hypoxia-inducible factor-1) in ccRCC cells and has significant impact on two relevant features of *VHL*-defective cells: the suppression of primary cilia that *in vivo* can lead to premalignant cysts and the increased motility that can lead to metastasis (168). However, the underlying mechanism by which VHL loss increases Aurora-A levels has not been clearly elucidated, although it has been suggested that HIF-1α mediates increased Aurora-A expression in VHL-null cells. Dere et al suggested that Aurora-A expression is driven by β-catenin transcription in the VHL null cells and that the level of Aurora-A was not modified by Hif1-α (169).

Clear cell renal cell carcinoma is also characterized by VHL inactivation and more recent data indicate that VHL interacts with primary cilia in renal epithelial cells. A hallmark of ccRCC is loss of the primary cilium. Loss of this key organelle in ccRCC is caused by loss of VHL and associated with increased Aurora-A and histone deacetylase 6 (HDAC6) activities, which drive disassembly of the primary cilium. Aurora-A is typically described as solely localized to the centrosome or centrosomally derived ciliary basal body and otherwise hard to detect in non-cycling normal mammalian cells. Evidence that HEF1/Aurora-A/HDAC6 signaling axis governs the resorption of cilia in addition to the previously defined roles for these proteins suggests a novel molecular mechanism to explain some cancer-associated ciliary loss. The focal adhesion protein HEF1 initially interacts with Aurora-A in G2 prior to the kinase activation. As the cell progresses throughout the cell cycle, focal adhesion disassembly releases a pool of HEF1, which promotes Aurora-A activation. Overexpression of HEF1 and Aurora-A promotes cytokinetic failure, and contributes to genomic instability. The protein association may interfere with a normal cellular interconversion between cilia and centrosome and contributes to cell cycle by staging critical signaling complexes that govern emergence from quiescence to S phase, and initiation of M phase.

**Figure 2:**
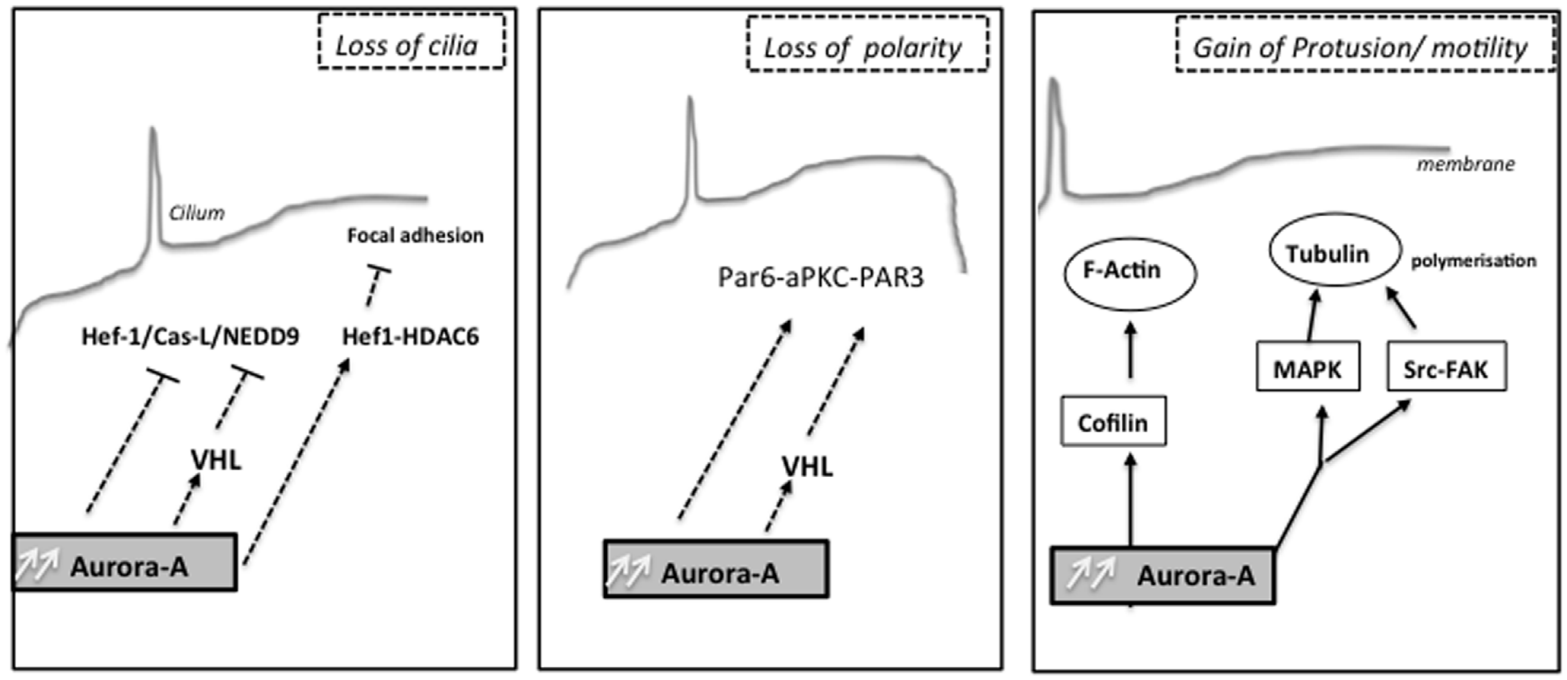
**Non-mitotic functions of the kinase Aurora-A: different biological processes as cilia resorption, cell polarity or cell migration are influenced by Aurora-A upstream mitosis, which can later affect chromosome segregation**.

Interestingly, formation of renal cysts is very strongly linked to defects in planar cell polarity control (170, 171). Aurora-A has also been found to directly phosphorylate Par-6, which together with atypical PKC and Par-3 regulate asymmetric cell division and cell polarity (172) and the changes in Ca^2+^ signaling induced by autosomal-dominant PKD (ADPKD)-associated mutations in the *PKD1* and *PKD2* genes (173–175). Low concentrations of drugs that inhibit Aurora-A activity raise basal intracellular Ca^2+^ levels in renal cells and PC2-dependent Ca^2+^ release. It has been also demonstrated that Aurora-A directly binds and phosphorylates PC2, and consequently may provide a mechanism by which Aurora-A inhibition limits PC2 Ca^2+^ channel activity. Moreover, the release of Ca^2+^ from the ER to the cytoplasm transiently activated Aurora-A, based on induced direct Ca^2+^-calmodulin (CaM) binding to Aurora-A. The non-mitotic activities of Aurora-A likely contribute to deregulation of growth in tumor cells overexpressing Aurora-A (Figure 2).

## Conclusion

Clear cell renal cell carcinoma is the predominant type of kidney cancer. ccRCC develops in the renal proximal tube and is linked to biallelic inactivation of the VHL tumor suppressor gene. Mitotic kinases defects mostly lead to aneuploid tumors and the sustained overexpression and activity of various mitotic kinases, including Aurora-A, Polo-like (Plk1), CK2, DCLK in diverse human tumors strongly indicate that these entities are intimately involved in the development of errors in chromosome segregation. Nevertheless, the non-mitotic functions of these kinases involved in the process of ciliogenesis, hypoxia, the EMT as well as the cell polarity likely play an important role in the process of tumorigenesis in kidney cancer as they also lead to genetic instability.

## Conflict of Interest Statement

The authors declare that the research was conducted in the absence of any commercial or financial relationships that could be construed as a potential conflict of interest.
